# Circulating interleukin-33 levels in obesity and type 2 diabetes: a systematic review and meta-analysis

**DOI:** 10.1152/ajpendo.00157.2024

**Published:** 2024-08-22

**Authors:** Ghalia Missous, Nicholas Van Panhuys

**Affiliations:** Laboratory of Immunoregulation, Disease Modelling and Therapeutics Department, Sidra Medicine, Doha, Qatar

**Keywords:** inflammation, interleukin-33, metabolic disorders, obesity, type 2 diabetes

## Abstract

Obesity and type 2 diabetes (T2D) are increasingly prevalent worldwide, and there is a critical need for novel interventions. Interleukin-33 (IL-33), an anti-inflammatory cytokine that regulates metabolism, is a promising biomarker for these conditions. The goal of this systematic review and meta-analysis is to examine the role of IL-33 in obesity and T2D, assessing its potential in predicting disease progression. A systematic search was performed on Scopus, Web of Science, and PubMed up until May 30, 2023. Each study was assessed for quality and sources of bias using the relevant critical appraisal checklists. Meta-analyses were conducted to compare IL-33 levels in individuals with obesity and T2D versus healthy controls (HC), and in obesity alone versus HC. Eighteen studies were included in the systematic review, and nine qualified for meta-analyses. The analyses showed insufficient evidence to suggest a significant difference in IL-33 levels between individuals with T2D and HC (mean difference, MD = −79.95, 95% CI [−241.38; 81.48]), with substantial heterogeneity across the studies observed (*I*^2^ = 97.1%, τ^2^ = 33,549.15). Similarly, there was insufficient evidence to suggest a significant difference between nondiabetic individuals with obesity and HC (MD = −7.31, 95% CI [−25.74; 11.13]), and heterogeneity was noted (*I*^2^ = 86.2%, τ^2^ = 342.45). There is insufficient evidence to indicate significant differences in IL-33 levels in individuals with T2D or obesity compared with HC. The results suggest a need for improved IL-33 measurement methods to reduce heterogeneity, enhancing understanding of the role of IL-33 in obesity and T2D, and informing future research and therapeutic strategies.

**NEW & NOTEWORTHY** Our research finds an inconclusive relationship between IL-33 serum levels in individuals with type 2 diabetes (T2D) and nondiabetic individuals with obesity. In addition, we note a potential gender association with IL-33 serum levels. Further studies with larger cohorts are required to assess the significance of serum IL-33 in T2D and obesity. Urgent standardization is needed in IL-33 quantification and reporting methods for reliable comparisons.

## Graphical abstract

**Figure:**
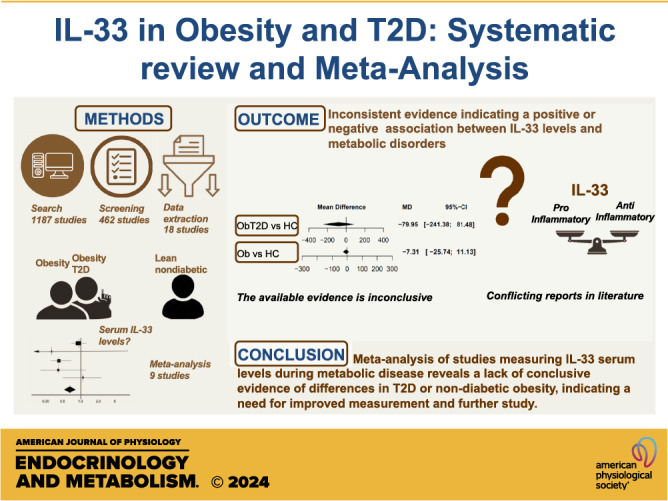

## INTRODUCTION

Considering the substantial impact of obesity and type 2 diabetes (T2D) on global health (1, 2), it has become imperative to identify effective interventions and explore novel treatment strategies for these diseases. The interleukin-1 cytokine family contains key inflammatory mediators, including IL-1α and IL-1β. These cytokines have garnered significant attention due to their roles in promoting the metabolic dysfunction and inflammation associated with obesity, which consequently contributes to the development of insulin resistance and T2D (3). In comparison, interleukin-33 (IL-33), which is also a member of the interleukin-1 family, has attracted considerable interest as a potential therapeutic agent, and a growing body of research over the past two decades has focused on its ability to promote homeostasis and induce thermogenic effects and beiging in adipose tissue (4). IL-33 has the potential to influence the interplay between inflammation and metabolism, a key factor in the development of T2D among individuals with obesity (5). Given that chronic low-grade inflammation is a hallmark of metabolic diseases, IL-33 has the potential to be used as a biological agent due to its role as an immunoregulatory cytokine with the capacity to suppress inflammatory responses (6).

Animal models have established a metabolically protective role for IL-33 in adipose tissue under homeostatic conditions (7) and have confirmed its mechanism as a key regulator of brown adipose tissue (8). A developmental feature, that is predominant in newborn mammals, is responsible for thermogenesis via the metabolism of stored lipids. Moreover, increased levels of IL-33 have been demonstrated to promote the browning of white adipose tissue (WAT) (9), and in an obese diabetic mouse model, treatment with IL-33 resulted in significant weight loss, decreased blood glucose levels, and improved glucose and insulin tolerance (10).

Recent human studies have further highlighted the potential significance of IL-33 in the context of obesity and T2D. IL-33 plays a pivotal role in immune regulation and maintenance of WAT homeostasis, where it is produced by a variety of cells, including preadipocytes, epithelial cells, endothelial cells, and fibroblasts (11). Recent research has indicated a negative correlation between IL-33 levels and body weight in overweight people, where it was observed to confer a protective lipid/metabolic profile in nondiabetics (6). Conversely, in individuals with obesity, an increased expression of IL-33 within the cell lining of adipose tissue has been observed (12). Whereas, in the context of T2D, researchers have noted reduced IL-33 expression in the adipose tissue of patients with both prediabetes and type 2 diabetes compared with those without metabolic disease (13).

Given the progression from obesity to T2D, understanding IL-33’s role across the spectrum of disease is crucial. Investigating IL-33 in obese individuals, both with and without T2D, can provide valuable insights into how IL-33 levels change as obesity progresses to T2D. To identify early biomarkers and potential intervention points to prevent disease progression.

This systematic review and meta-analysis represents the first comprehensive evaluation of reported serum IL-33 levels in individuals with obesity and T2D. Despite the increasing volume of research on IL-33, there remains a critical need to consolidate and critically appraise the existing evidence to bridge knowledge gaps and offer more thorough insight into the mechanisms underlying obesity and T2D.

## MATERIALS AND METHODS

### Literature Search and Study Selection

The study protocol received official registration in the Prospective Register of Systematic Reviews (PROSPERO) with the registration identification number CRD42024471632. Following the Preferred Reporting Items for Systematic Reviews and Meta-Analyses (PRISMA) guidelines (14), we conducted a comprehensive search of Scopus, PubMed, and Web of Science databases. Our search strategy, from inception until May 30, 2023, included both text words and Medical Subject Headings (MeSH) terms using Boolean logic operators: (“Interleukin-33” OR “Interleukin33” OR “IL-33” OR “IL33”) AND (“type 2 diabetes” OR “T2D” OR “diabetes” OR “diabetic” OR “metabolic syndrome” OR “obesity” OR “obese”). We also screened the reference lists of relevant publications for potentially eligible studies. The titles and abstracts were screened using Rayyan (http://rayyan.qcri.org) (15). The search and screening were independently conducted by both authors, and there were no discrepancies.

The research question focused on elucidating the significance of IL-33 levels in individuals with metabolic disorders, specifically focusing on obesity and T2D. This investigation involved evaluating serum IL-33 levels and comparing them among individuals with T2D combined with obesity, individuals with nondiabetic obesity, and healthy controls (HC). Although no age restriction was applied in the research, to be included, articles involved adult human participants (18 yr or older) with obesity, with or without T2D. Studies were excluded if they were animal studies, type 1 diabetes studies, case reports, opinion reports, descriptive papers, letters to the editor, reviews, or protocols. Unpublished data and non-English reports were also excluded (16).

### Data Extraction

A predefined extraction table was used to extract and compile relevant datasets, such as author and publication year, country, study design, participant characteristics, and potential confounders. For studies included in the meta-analyses, additional data, including mean serum IL-33 levels and measurement methods, were extracted.

For the meta-analyses, we retrieved data on serum IL-33 levels (means ± SD) measured in individuals with T2D and/or obesity, and data from healthy controls. In cases of missing data, efforts were made to communicate with the respective authors for clarification. Of all the authors contacted two responded: one author provided the required data (17), whereas another indicated that the data were unavailable (6). In the latter case, data extraction was performed from the graphical representations.

The presentation of serum IL-33 data varied across the studies. Two studies displayed data graphically without providing exact numerical values: one used the means ± SE, and the other presented median (minimum-maximum) values (6, 18). In addition, one study reported data as the geometric mean (range) (19), whereas two studies presented data as the means ± SE (20, 21). To extract information from the graphs, we used WebPlotDigitizer 4.6. This tool has demonstrated a high level of accuracy and consistency in extracting data from graphical representations (22, 23).

The medians (minimum-maximum) and geometric means (range) were transformed into means ± SDs using well-recognized and validated methods (24, 25), as described in Supplemental File S1.

### Quality Assessment and Risk of Bias

The methodological quality and risk of bias were evaluated using Joanna Briggs Institute (JBI) critical appraisal checklists tailored to specific study designs (26, 27). These checklists served a dual purpose, as they were used both for quality assessment and risk of bias evaluation. Recognizing the inherent link between methodological quality and the potential for bias, these checklists facilitate a comprehensive examination of each study’s design, conduct, and analysis (28). Responses in these checklists are categorized as “yes,” “no,” “unclear,” or “not applicable.” The overall methodical quality was categorized as follows: high (***), if the majority of criteria were met with minimal or no susceptibility to bias; acceptable (**), if most criteria were met, albeit with some flaws that could introduce a potential risk of bias; or low (*), if most criteria were not met, signifying deficiencies in meeting essential elements of the study design.

### Statistical Analysis

The meta package in RStudio (v.2023.12.0 + 369) was used for the statistical analysis. The mean difference (MD) was chosen as the measure of effect, and the pooled MD was calculated using random effects models to account for potential heterogeneity in effect sizes across studies (29). The heterogeneity of the studies was measured using the inconsistency index *I* square (*I*^2^) and Tau squared (τ^2^). τ^2^ reflects the amount of true heterogeneity, whereas *I*^2^ quantifies the degree of heterogeneity among effect sizes of the included studies. It provides an estimate of the proportion of total variation in effect sizes that is due to true heterogeneity rather than random chance. *I*^2^ values of 25, 50, and 75% corresponded to low, medium, and high heterogeneity, respectively (30). The meta-analysis results were visually presented using forest plots.

### Sensitivity Analysis

We conducted sensitivity analyses to assess the robustness of our findings. This process involved leave-one-out analyses, systematically excluding individual studies to evaluate their impact on the pooled MD. In addition, we performed sensitivity analyses using a fixed-effects model to assess the influence of the statistical model on the overall meta-analytic outcome.

## RESULTS

### Characteristics of the Included Studies

**Figure 1.**
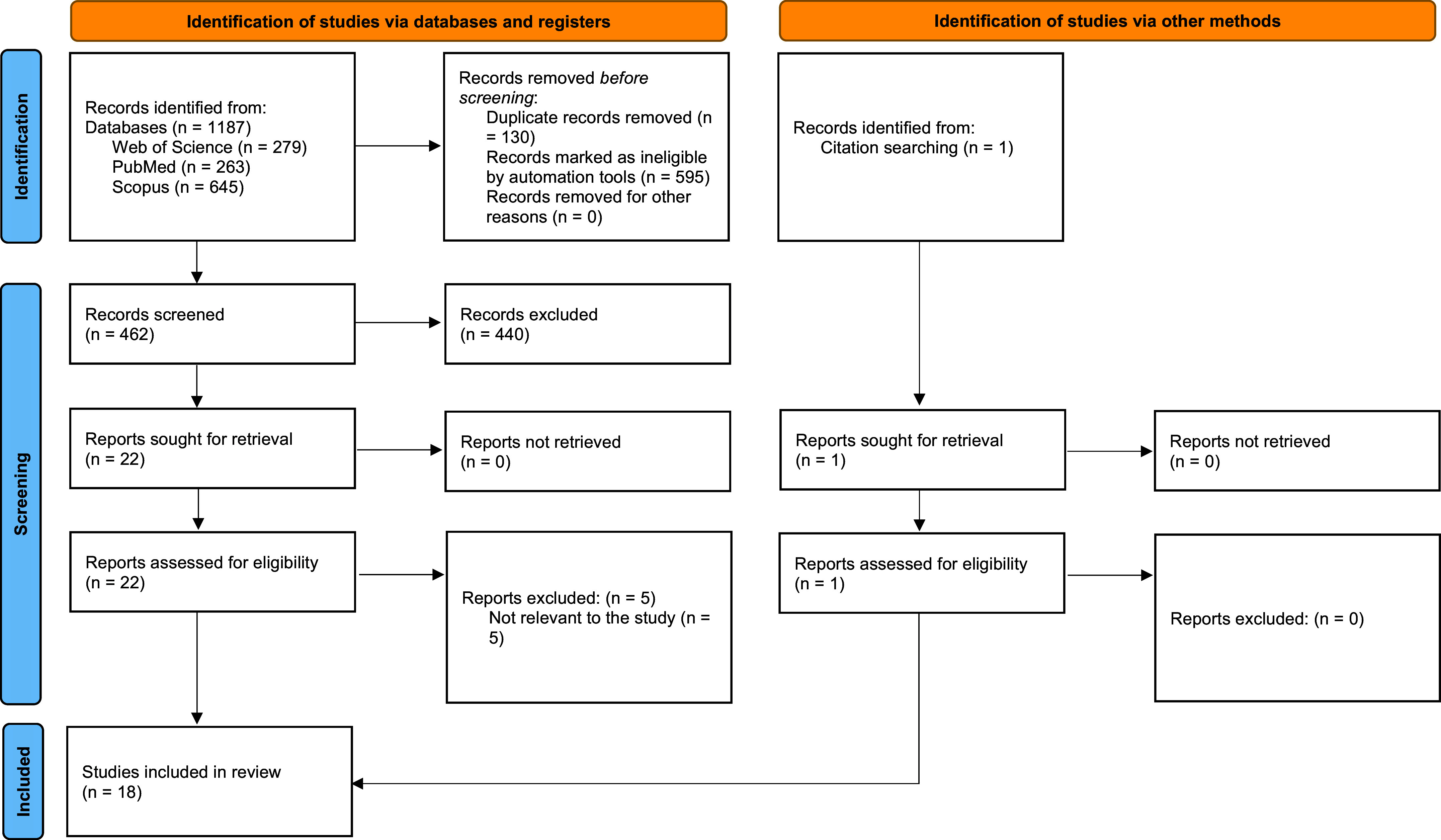
PRISMA flow chart of study selection process. PRISMA, Preferred Reporting Items for Systematic Reviews and Meta-Analyses.

According to the PRISMA 2020 guidelines (14), Fig. 1 depicts a graphical representation of the systematic study selection process. A total of 462 studies were identified. These studies were subjected to a thorough screening procedure, resulting in the inclusion of 18 studies.

The characteristics of these studies are summarized in Table 1. Additional data and measurement methods for the studies selected for meta-analysis are summarized in Table 2. The aims and key findings of the studies selected for the narrative synthesis are presented in Table 3, and those selected for the meta-analysis are presented in Table 4.

**Table 1.** Characteristics of studies included in the systematic review and meta-analyses

			Disease Group				Control Group			
Authors/Year Country (Reference)	Study Design	Disease/Intervention	Sample Size	Age	Male/Female	BMI	Sample Size	Age	Male/Female	BMI
Alfadul et al. (2023) Saudi Arabia (17)	Cross-sectional	PD T2D	PD: 88 T2D: 121	PD: 42.3 ± 8.8 T2D: 44.0 ± 8.7	PD: 38/50 T2D: 6/95	PD: 32.7 ± 5.9 T2D: 32.6 ± 7.2	Healthy controls: 198	39.3 ± 8.9	87/111	28.7 ± 5.3
Anand et al. (2014) India (19)	Cross-sectional	T2D Diabetic nephropathy	T2D: 79 DN: 82	T2D: 54.7 ± 13.4 DN: 56.3 ± 10.9	T2D: 49/30 DN: 46/36	T2D: 25.2 ± 4.2 DN: 27.4 ± 5.6	Normal glucose tolerance group: 61	46.2 ± 16	31/30	22.9 ± 5.0
Beneventi et al. (2020) Italy (31)	Case-control study	T2D Preeclampsia	Overweight/Obesity normotension: 18 Overweight/Obesity hypertension: 17	34 (31–36)	All females	≥24.9	Controls normotension: 42 Controls Hypertension: 5	33.5 (29.7–38)	All females	<24.9
Bonfante et al. (2022) Brazil (32)	RCT	T2D/Combined training	Combined Training Group with T2D: 15	40–60	6/9	29.61 ± 3.60	Control group with T2D: 17	40–60	9/8	29.61 ± 3.60
Çakıcı et al. (2021) Turkey (33)	Cross-sectional	Obesity T2D	Nondiabetic obese: 25 Diabetic obese: 36	18–75	Not reported	33.75 ± 6.15	Healthy controls: 21	18–75	Not reported	23.44 ± 0.84
de Lima Azambuja et al. (2015) Brazil (34)	Cross-sectional	Asthma Obesity	Overweight: 25 Obese: 25	Overweight: 57.6 ± 15.6 Obese: 52.8 ± 14.6	Overweight: 7/18 Obese: 7/18	Overweight: 25.0–29.9 Obese: ≥ 30	Eutrophic: 25	49.0 ± 19.9	7/18	18.5–24.9
Demyanets et al. (2020) Austria (35)	Prospective cohort study	Morbid obesity T2D/bariatric surgery	Obese T2D: 25 Obese PD: 14	Entire cohort 40.8 ± 12.7	Entire 24/56	Entire cohort 44.2 ± 3.9	Obese with Normal glucose tolerance: 62	Entire cohort 40.8 ± 12.7	Entire cohort 24/56	Entire cohort 44.2 ± 3.9
Dorneles et al. (2019) Brazil (36)	RCT	High-intensity interval training	Obese nonexercisers: 20 Obese moderately trained: 15 Obese intensely trained: 10	33.3–34.9	All males	31–32.7	Lean nonexercisers: 15 Lean moderately trained: 20 Lean Intensely trained: 10	33.3–34.9	All males	23.3–23.7
Gruzdeva et al. (2018) Russia (37)	Case-control study	Obesity MI	Visceral obesity with MI: 59 No visceral obesity with MI: 29	Visceral obesity: 58.50 (53.0–63.0) No visceral obesity: 56.0 (51.5–63.5)	65/23	Visceral obesity 28.7 (171–39.1) No visceral obesity 25.9 (18.3–38.4)	Healthy controls: 30	58.42 (52.2–71.1)	Not reported	Not reported
Hasan et al. (2014) Kuwait (6)	Cross-sectional	T2D	T2D lean: 1 T2D overweight: 2 T2D obese: 12	49.5 ± 11.4	Not reported	33 ± 3.9	Nondiabetic lean: 10 Nondiabetic overweight:8 Nondiabetic obese: 13	38.7 ± 11.35	Not reported	28.54 ± 5.3
Hasan et al. (2019) Kuwait (13)	Cross-sectional	T2D	Prediabetic: 23 T2D: 47	PD: 46 ± 12.1 T2D: 54 (23, 72)	Not reported	PD: 31.16 ± 5.54 T2D: 31.3 ± 3.79	Normoglycemia: 21	39 ± 11	Not reported	30.1 ± 5.2
Katsogiannos et al. (2021) Sweden (20)	Prospective cohort study	Obesity and T2D/bariatric surgery	Obese T2D: 21 Obese without T2D: 13	Obese T2D: 49 ± 2 Obese without T2D: 42 ± 3	Obese T2D: 3/18 Obese without T2D: 1/12	Obese T2D: 38.3 ± 0.9 Obese without T2D: 43.1 ± 0.8	Healthy controls: 25	39 ± 3	11/14	24.4 ± 0.5
Liu et al. (2015) China (38)	RCT	T2D/combined training	T2D conventional therapy group: 20 T2D intensive therapy group: 22	Not reported	T2D: 12/30	T2D: 52.59 ± 11.43	Healthy controls: 20	Not reported	7/13	51.20 ± 11.34
Nesic et al. (2022) Serbia (39)	Cross-sectional	Metabolic syndrome	Metabolic syndrome: 67	41.69 ± 15.55	29/38	32.93 ± 5.58	Healthy controls: 45	33.08 ± 15.14	19/26	23.16 ± 3.70
Pereira et al. (2023) Sweden (40)	Cross-sectional	T2D	Cohort 1 T2D: 20 Cohort 2 T2D: 11	Cohort 1 T2D: 60 (52–65) Cohort 2 T2D: 54 (43–59)	Cohort 1 T2D: 10/10 Cohort 2 T2D: 2/9	Cohort 1 T2D: 30.06 (26.86–34.32) Cohort 2 T2D: 37.3 (32.5–39.2)	Healthy controls Cohort 1: 20 Cohort 3: 11 Cohort 2: 9	Cohort 1: 60 (52–68) Cohort 2: 59 (51–63) Cohort 3: 25 (21–31)	Cohort 1: 10/10 Cohort 2: 3/6 Cohort 3: 7/4	Cohort 1: 30.7 (28.2–34.6) Cohort 2: 28.6 (25.1–30.8) Cohort 3: 22.6–30.7
Singh et al. (2023) India (21)	Case-control study	T2D	T2D: 30	Not reported	Not reported	Not reported	Healthy controls: 30	Not reported	Not reported	Not reported
Tang et al. (2021) China (5)	Cross-sectional	Obesity Metabolic disorders	MUOO: 72 MHOO: 72	MUOO: 44.82 (12.72) MHOO: 44.39 (12.74)	MUOO: 48/24 MHOO: 49/23	MUOO: 28.15 (2.71) MHOO: 27.38 (2.48)	Healthy controls: 73	44.48 (12.05)	48/25	21.99 (1.50)
Zeyda et al. (2013) Austria (18)	Cross-sectional	Severe obesity	Severely obese: 20	Not reported	04/16	53.0 ± 2.5	Lean to pre-obese/overweight control: 20	Not reported	Matching obese group	25.2 ± 0.7

Data are presented as the means ± SD, median (interquartile range), or range: years for age, kg/m^2^ for BMI. BMI, body mass index; DN, diabetic nephropathy; MHOO, metabolically healthy overweight/obese; MI, myocardial infarction; MUOO, metabolically unhealthy overweight/obese; PD, prediabetes; RCT, randomized controlled trial; T2D, type 2 diabetes.

**Table 2.** Studies included in the meta-analysis

Authors/Year (Reference)	Comparison Group	IL-33 Measurement Assay	Serum IL-33 Levels in the Disease Group	Serum IL-33 Levels in the Healthy Control Group
Alfadul et al. (2023) (17)	T2D vs. Healthy controls	ELISA, R&D Systems	Means ± SD 2.87 ± 1.38 pg/mL	Means ± SD 2.78 ± 1.54 pg/mL
Anand et al. (2014) (19)	T2D vs. Healthy controls	ELISA, eBioscience	Geometric mean (range) 118.6 (72.6–193.7) pg/mL	Geometric mean (range) 348.0 (318.2–380.6) pg/mL
Singh et al. (2023) (21)	T2D vs. Healthy controls	DuoSet ELISA, R&D Systems	Means ± SE T2D: 40 ± 7 pg/mL	Means ± SEM 458 ± 112 pg/mL
Çakıcı et al. (2021) (33)	T2D vs. Healthy controls Obesity vs. Healthy controls	ELISA, CUSABIO	Means ± SD T2D: 5.70 ± 2.02 pg/mL Obesity: 5.43 ± 2.04 pg/mL	Means ± SD 5.22 ± 1.58 pg/mL
Katsogiannos et al. (2021) (20)	T2D vs. Healthy controls Obesity vs. Healthy controls	Magnetic bead-based Luminex assay, R&D Systems	Means ± SE T2D: 5.9 ± 1.0 pg/mL Obesity: 10.4 ± 3.9 pg/mL	Means ± SE 5.6 ± 0.8 pg/mL
Tang et al. (2021) (5)	Obesity vs. Healthy controls	ELISA, CUSABIO	Means ± SD MHOO: 80.46 ± 26.65 pg/mL	Means ± SD 76.24 ± 21.42 pg/mL
Dorneles et al. (2019) (36)	Obesity vs. Healthy Controls (Nonexercisers group)	ELISA	Means ± SD 143.2 ± 21.5 pg/mL	Means ± SD 179.7 ± 16.5 pg/mL
Hasan et al. (2014) (6)	Obesity vs. Healthy controls	DuoSet ELISA, R&D Systems	Median (minimum to maximum) 90.98 (31.37–346.66) pg/mL Extracted from a graph	Median (minimum to maximum) 162.35 (65.09–531.76) pg/mL Extracted from a graph
Zeyda et al. (2013) (18)	Obesity vs. Healthy controls	ELISA, Apotech/Enzo Life Sciences	Means ± SE 440.23 ± 139.56 pg/mL Extracted from a graph	Means ± SE 510.05 ± 88.77 pg/mL Extracted from a graph

Data are presented as the means ± SD, the means ± SE, or median (minimum to maximum). ELISA, enzyme-linked immunosorbent assay; IL-33, interleukin-33; MHOO, metabolically healthy overweight/obese; SD, standard deviation; SE, standard error of the mean; T2D: type 2 diabetes.

**Table 3.** Aims and key findings of studies selected for the narrative synthesis

Study	Main Aim	Key Findings	Notes/Considerations
Beneventi et al. (2020) (31)	Explore the correlation between BMI and IL-33 and its potential implications for pregnancy	- Correlation between BMI and IL-33 suggests a potential link between IL-33 and adiposity	- Pilot study
		- Lower serum IL-33 concentrations in women with preeclampsia	- Small sample size
Bonfante et al. (2022) (32)	Investigate chronic and acute training responses in overweight with type 2 diabetes, focusing on serum prothermogenic/anti-inflammatory factors	- Improved metabolic patterns and physical training positively influenced IL-33 and FNDC5/irisin concentrations, especially during fasting	- Further exploration needed regarding long-term effects
		- Exercise-induced changes in IL-33 levels suggest a link between physical activity, metabolic health, and anti-inflammatory responses in T2D	
de Lima Azambuja et al. (2015) (34)	Investigate the relationship between asthma and obesity, examining the roles of adiponectin, CRP, and IL-33	- IL-33 may have opposing effects in asthma and obesity, inducing Th2 responses and exhibiting protective effects in obesity-related inflammation	- Further clarification needed regarding the impact of IL-33 on asthma
		- IL-33 levels in asthmatic patients with different BMIs indicated lower levels in patients with obesity, although not statistically significant	
Demyanets et al. (2020) (35)	Investigate circulating levels of sST2, a decoy receptor for IL-33, in patients with obesity before and after bariatric surgery	- sST2 levels decreased after surgery, with a more pronounced decrease in patients with diabetes	- The importance of distinguishing different forms of IL-33 was highlighted
		- Associations between sST2 levels and metabolic factors observed before surgery did change postsurgery	
		- IL-33 levels did not significantly change postsurgery	
Gruzdeva et al. (2018) (37)	Examine IL-33 levels during MI, hospitalization, and its correlation with epicardial adipose tissue thickness.	- IL-33 levels increased during MI, positively correlating with epicardial adipose tissue thickness	- A relationship between IL-33 levels and cardiac fibrosis was observed
		- IL-33 exhibited antihypertrophic and antifibrotic effects, suggesting a protective role against cardiac fibrosis	
		- Elevated IL-33 in patients with MI without visceral obesity associated with limited cardiac fibrosis	
Hasan et al. (2019) (13)	Explore the expression levels of IL-33 and ST2 in adipose tissue and their associations with metabolic parameters	- IL-33 expression was notably lower in individuals with prediabetes and T2D compared with normoglycemic individuals	- Dysfunctional IL-33/ST2 axis in prediabetes was suggested
		- IL-33 expression negatively correlated with HbA1c levels and positively correlated with beiging adipose tissue markers	
Liu et al. (2015) (38)	Investigate the effects of combined aerobic and resistance training on glycolipid metabolism and inflammation levels in patients with T2DM	- Exercise training significantly improved glycolipid metabolism and reduced low-grade inflammation in patients with diabetes	- Small sample size
		- Intensive therapy group had significantly higher serum IL-33 levels after exercise training compared with conventional therapy group	- Further experiments needed
		- No significant differences in IL-33 levels between exercise and control groups were reported	
Nesic et al. (2022) (39)	Investigate the association between adiponectin and interleukin-33 in patients with metabolic syndrome	- Patients with low adiponectin had less pronounced characteristics of metabolic syndrome with significantly higher values of IL-33 compared with patients with high adiponectin	- Future clinical studies needed for confirmation
		- IL-33 and adiponectin might contribute to the development of metabolic syndrome	
Pereira et al. (2023) (40)	Investigate the involvement of IL-33 in adipose tissue metabolism and its association with insulin resistance and type 2 diabetes	- IL-33 gene expression was higher in subcutaneous adipose tissue of patients with T2D compared with healthy controls	- Positive correlation between ST2 expression and BMI raises questions about the potential influence of BMI on IL-33 effects
		- IL-33 mRNA expression positively correlated with markers of dysglycemia, insulin resistance, and adiposity	
		- IL-33 may play a role in the development of insulin resistance and T2D by inhibiting glucose uptake in adipocytes	

BMI, body mass index; MI, myocardial infarction; sST2, soluble ST2; T2D, type 2 diabetes.

**Table 4.** Aims and key findings of the studies included in the meta-analysis

Study	Main Aim	Key Findings	Notes/Considerations
Alfadul et al. (2023) (17)	Investigate NLRP3 inflammasome with interleukins in PD and type 2 diabetes	- NLRP3 expression is significantly influenced by T2DM status and age	- Higher levels of NLRP3 were observed in the female PD group, suggesting a potential gender-specific association
		- It is associated with IL-18, IL-1α, and IL-33 levels	
		- Positive correlation between NLRP3 and IL-33	
Anand et al. (2014) (19)	Explore cytokine levels in DN and study the role of IL-33	- Decreased IL-33 in diabetic nephropathy	- IL-33 potential therapeutic target for DN
		- Serial decline with insulin resistance and microalbuminuria	
Çakıcı et al. (2021) (33)	Investigate the relationship between IL-33 and TF in patients with nondiabetes and diabetes with obesity	- No significant difference in IL-33 levels between groups	- TF may serve as a prognostic marker for diabetes in the context of obesity
		- IL-33 positively correlated with metabolic disorders	- Further experiments needed
		- IL-33 modulates TF expression and activity	
Dorneles et al. (2019) (36)	Examine effects of HIIT on the immune system in sedentary men with obesity	- Elevated IL-33 in moderate and high cardiorespiratory fitness	- Potential benefits of exercise in modulating inflammation in both lean and obese individuals highlighted
		- One-week HIIT increases Treg cells and IL-10 in individuals with obesity	
Hasan et al. (2014) (6)	Investigate the relationship between IL-33 and metabolic abnormalities in obesity	- IL-33 reduced in nonlean subjects	- Small sample sizes
		- IL-33 correlated with lipid profile and associated with glucose regulation	
Katsogiannos et al. (2021) (20)	Study cytokine and adipokine changes after gastric bypass surgery	- IL-33 levels higher in obesity	- IL-33 suggested as a marker of obesity-related inflammation
		- No significant change post-surgery	
Singh et al. (2023) (21)	Evaluate IL-33 and sST2 in patients with T2D with or without metabolic syndrome	- Protective role of IL-33 on β-cell mass	- Missing anthropometric and demographic data (BMI, age, gender)
		- Lower IL-33 in patients with T2D	- Small sample size
Tang et al. (2021) (5)	Investigate the correlation between IL-33 levels and metabolic phenotypes of obesity	- Higher IL-33 in overweight/obese individuals	- Insulin levels and Th1 cytokine levels were not measured
		- Positive correlation with metabolic syndrome risk factors	
Zeyda et al. (2013) (18)	Study alterations in obesity and the role of IL-33 in adipose tissue inflammation	- Elevated IL-33 in omental and subcutaneous adipose tissue of humans with severe obesity	- Small cohort size
		- IL-33 primarily expressed in the vasculature of human adipose tissue, with endothelial cells identified as the main source	
		- Protective effects of IL-33 in obesity	

BMI, body mass index; DN, diabetic nephropathy; HIIT, high-intensity interval training; NLRP3, nucleotide-binding domain, leucine-rich repeat family pyrin domain containing 3; PD, prediabetes; sST2, soluble ST2; T2D, type 2 diabetes; TF, tissue factor.

### Quality Assessment and Risk of Bias

Most studies were rated as high quality, indicating minimal or no susceptibility to bias. Two studies received an acceptable quality rating (21, 32), indicating the presence of minor flaws associated with a potential risk of bias. Importantly, none of the included studies were categorized as low quality. All studies adhered to or surpassed the criteria outlined in materials and methods (Supplemental File S2).

### Meta-Analysis

Nine relevant studies with comparable study populations were chosen for meta-analysis (Table 2). The inclusion criterion was that only studies meeting the data pooling and quantitative analysis requirements were included. The forest plots display confidence intervals (CIs = 95%) along the horizontal axis, and black squares represent the point estimates for each study on the plot. The central vertical line signifies a state of no effect.

### IL-33 Serum Levels in Patients with T2D Versus Healthy Controls

**Figure 2.**
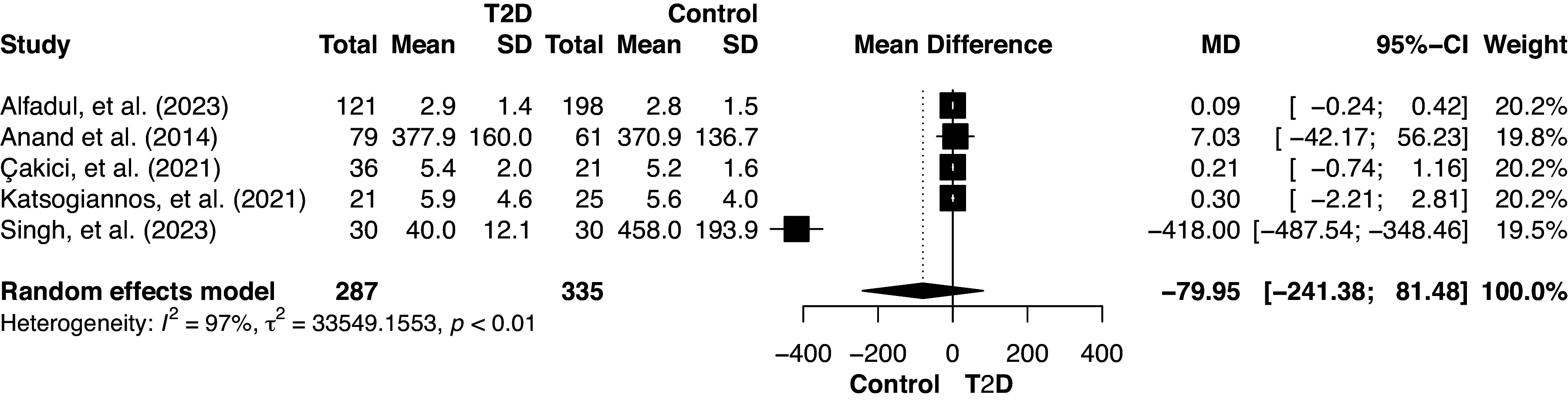
Forest plot of serum IL-33 (pg/mL) in individuals with type 2 diabetes (T2D) vs. healthy controls.

Five studies (17, 19–21, 33) involving 622 patients reported IL-33 serum levels in patients with T2D with obesity and healthy controls. According to the random-effects model, the overall effect size was −79.95 (95% CI [−241.38; 81.48], *P* = 0.33), suggesting insufficient evidence to support a significant impact of obesity-related T2D on IL-33 levels. However, substantial heterogeneity across the studies was observed (*I*^2^ = 97.1%). The significant *P* value of the heterogeneity test (<0.001) indicated that the observed heterogeneity was unlikely to be attributable to chance (Fig. 2). A negative MD suggested a tendency toward higher IL-33 levels in healthy controls, but the wide confidence interval and the presence of substantial heterogeneity highlighted uncertainty in the estimate. Data from Singh et al. (21) showed a strong negative MD, indicating significantly lower IL-33 levels in individuals with T2D.

### IL-33 Serum Levels in Nondiabetic Individuals with Obesity Versus Healthy Controls

**Figure 3.**
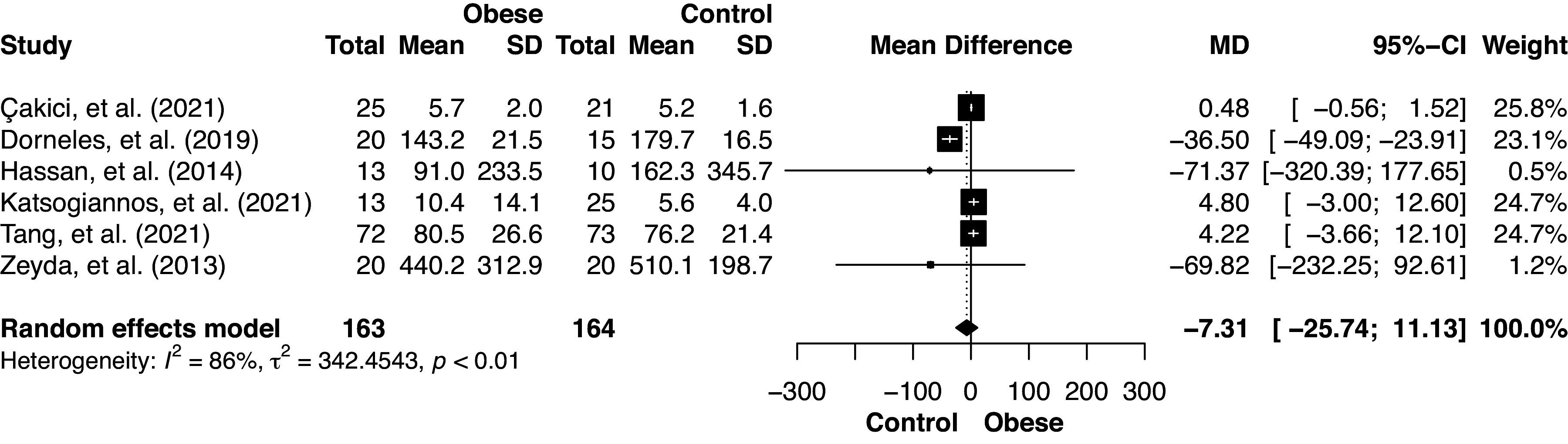
Forest plot of serum IL-33 (pg/mL) in individuals with obesity vs. healthy controls.

Serum IL-33 levels were compared between nondiabetic individuals with obesity and healthy controls across six studies (5, 6, 18, 20, 33, 36), involving a total of 327 patients (Fig. 3). Using the random-effects model, effect size revealed an MD of −7.31 with a 95% CI of [−25.74; 11.13], indicating that the overall effect did not reach statistical significance. A substantial *I*^2^ value of 86.2% suggested significant heterogeneity among studies.

### Sensitivity Analysis

Table 5 presents the results of the sensitivity analysis examining the impact of excluding individual studies on the meta-analytical findings of serum IL-33 levels in individuals with T2D and obesity compared with healthy controls. Although not reaching statistical significance, the results consistently indicated a negative effect, suggesting a potential reduction in serum IL-33 levels among individuals with T2D and obesity compared with healthy controls. However, substantial heterogeneity across the remaining studies was still present. Notably, upon excluding the study by Singh et al. (21), the results showed a minimal positive MD with a narrow confidence interval and no observed heterogeneity. This implies that the inclusion of this study significantly contributed to the observed heterogeneity.

**Table 5.** Leave-one-out sensitivity analysis results for patients with T2D vs. healthy controls

Study	Pooled MD	95% CI	*I*²	τ^2^	Heterogeneity Test: *P*
T2D vs. healthy controls	−79.95	[−241.38; 81.48]	97.1%	33549.15	<0.001
Excluding Alfadul et al. (2023) (17)	−100.73	[−304.29; 102.82]	97.8%	42679.17	<0.0001
Excluding Anand et al. (2014) (19)	−102.06	[−303.1; 99.83]	97.8%	42135.45	<0.0001
Excluding Çakıcı et al. (2021) (33)	−100.77	[−304.30; 102.77]	97.8%	42670.85	<0.0001
Excluding Katsogiannos et al. (2021) (20)	−100.1	[−304.31; 102.73]	97.8%	42664.48	<0.0001
Excluding Singh et al. (2023) (21)	0.11	[−0.20; 0.41]	0.0%	0	0.9846

CI, confidence interval; MD, mean difference; T2D, type 2 diabetes.

**Figure 4.**
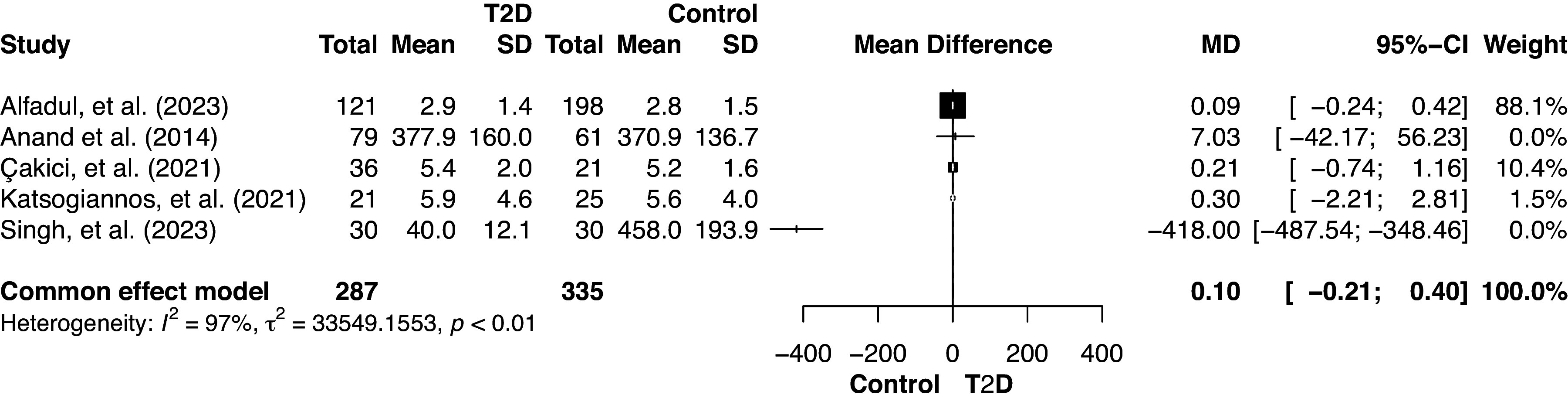
Serum IL-33 (pg/mL) in individuals with type 2 diabetes (T2D) vs. healthy controls using fixed-effects model.

The results of the sensitivity analyses using the fixed-effects model are shown in Fig. 4. The overall effect size was 0.10 (95% CI [−0.21; 0.40], *P* = 0.5315), indicating that there was not enough evidence for a significant effect. Substantial heterogeneity persisted (*I*^2^ = 97.1%), reflecting variability in the true effect size among studies. As depicted in Fig. 4, the data from Anand et al. (19) revealed a substantial positive MD of 7.03, indicating an increase in serum IL-33 levels among individuals with T2D. In contrast, data from Singh et al. (21) significantly deviated from the overall pattern, portraying a strongly negative MD of −418.00 and suggesting a decrease in IL-33 levels in patients with T2D.

The sensitivity analyses suggested that the results were robust. Both models consistently indicate a lack of sufficient evidence to reject the null hypothesis.

Sensitivity analyses of IL-33 serum levels in nondiabetic individuals with obesity versus healthy controls involving the exclusion of individual studies in the comparison group of individuals with obesity versus healthy controls revealed notable changes in the pooled MD and heterogeneity measures (Table 6). Upon excluding the study by Dorneles et al. (36), a distinct pattern emerged, revealing a pooled MD of 1.24 with a narrow 95% CI of [−1.34; 3.82]. Heterogeneity was markedly reduced to 0.0%, and the heterogeneity test yielded a nonsignificant *P* value of 0.5549. This implies that the inclusion of the study by Dorneles et al. (36) had a significant impact on the observed heterogeneity in the original analysis. It is important to note that this study exclusively involved male participants, which potentially serves as a confounding factor.

**Table 6.** Leave-one-out sensitivity analysis results for the obesity vs. healthy control comparison group

Studies	Pooled MD	95% CI	*I*²	τ^2^	Heterogeneity Test: *P*
Obese vs. healthy controls	−7.30	[−25.74; 11.13]	86.2%	342.45	<0.0001
Excluding Çakıcı et al. (33)	−10.78	[−36.32; 14.76]	88.6%	502.85	<0.0001
Excluding Dorneles et al. (36)	1.24	[−1.34; 3.82]	0.0%	1.87	0.5549
Excluding Hasan et al. (6)	−6.96	[−25.48; 11.55]	88.8%	343.78	<0.0001
Excluding Katsogiannos et al. (20)	−11.83	[−35.98; 12.32]	88.5%	450.83	<0.0001
Excluding Tang et al. (5)	−11.67	[−36.02; 12.68]	88.7%	459.01	<0.0001
Excluding Zeyda et al. (18)	−6.53	[−25.12; 12.058]	88.7%	344.05	<0.0001

CI, confidence interval; MD, mean difference.

**Figure 5.**
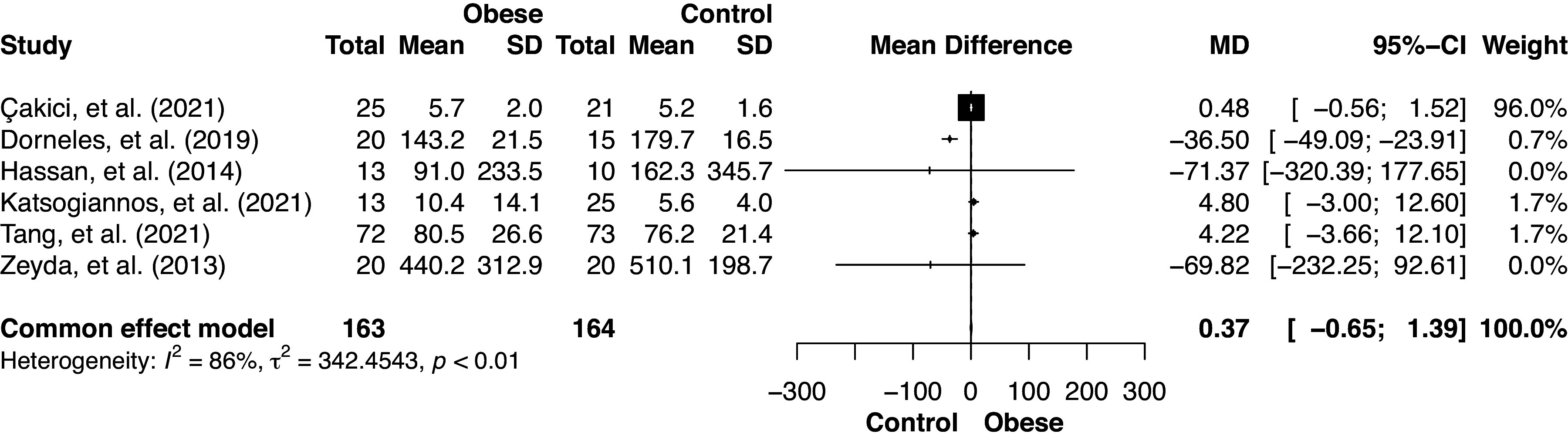
Serum IL-33 (pg/mL) in individuals with obesity vs. healthy controls using the fixed-effects model.

The fixed-effects model (Fig. 5) indicated a very small positive effect [MD = 0.37] with a 95% CI of [−0.65; 1.39], suggesting no statistically significant effect. An *I*^2^ value of 86.2% indicated a high degree of heterogeneity across the studies. The fixed-effects model indicated that studies by Çakıcı et al. (33), Katsogiannos et al. (20), and Tang et al. (5) provided more precise estimates with moderate-to-high weights, signifying their meaningful contribution to the overall meta-analysis. In comparison, studies by Dorneles et al. (36), Hasan et al. (6), and Zeyda et al. (18), with low or zero weightings, had less influence due to their lower precision. This is indicated by the wide confidence intervals around the population, which means following the application of a fixed effects model, which is indicative of a low degree of certainty about the true effect size.

The different methodological approaches consistently suggest that there is not enough evidence to reject the null hypothesis in the comparison of serum IL-33 levels between nondiabetic individuals with obesity and healthy controls.

## DISCUSSION

### Impact of IL-33: Insights from a Comprehensive Review of Studies

#### Adipose tissue, IL-33, and metabolic disorders.

The use of animal models to study the role of IL-33 in metabolic disease has revealed a protective role for IL-33, following the demonstration of anti-inflammatory and prohomeostatic properties within adipose tissue (41–43). In comparison, human studies exploring the role of IL-33 in adipose tissue have provided much more varied insights into the potential role of IL-33 in modulating the inflammatory processes that accompany the onset of metabolic disease. Elevated expression of IL-33 and its receptor ST2 has been reported in individuals with severe obesity, where increased levels were also associated with markers of inflammation (18). The authors also noted a localized increase of IL-33 expression in endothelial cells lining the blood vessels surrounding the adipose tissue. In comparison, serum levels of IL-33 did not differ between the study groups, suggesting that IL-33 production may be a localized response to obesity-related signals. Increased IL-33 expression levels have also been observed in the subcutaneous adipose tissue of patients with T2D both at the mRNA and protein levels (40). IL-33 levels showed a positive correlation with dysglycemia, insulin resistance, and adiposity markers and a negative correlation with gene pathways associated with glucose uptake and lipid storage. The effects of IL-33 on ex vivo glucose uptake by adipocytes were also assessed and shown to have a significant inhibitory effect, indicating a potential role for IL-33 in the development of insulin resistance. In contrast, a comparison of IL-33 and ST2 levels across individuals with normoglycemia, prediabetes, and T2D (13) showed an inverse association between IL-33 expression in adipose tissue and overall glycemia, suggesting a potential regulatory role of the IL-33/ST2 axis in glucose homeostasis. A positive correlation between IL-33 and immunogenic markers was also highlighted in this study that is indicative of a potential role for IL-33 as a mediator between the immune response and metabolic dysfunction. The discordance between these two studies may potentially be explained by IL-33 exerting differential effects dependent on the stage of glucose dysregulation, though this remains to be established.

In addition to the analysis of IL-33 expression in adipose tissue, serum IL-33 levels have been reported to be significantly reduced in patients with T2D, irrespective of the presence of symptomatic metabolic syndrome (21). Here, the levels of IL-33 in patients with T2D with either controlled or uncontrolled metabolic syndrome were compared with normoglycemic controls, and it was observed that IL-33 was significantly decreased in both T2D groups, whereas ST2 was significantly increased. As adiponectin has previously been shown to exert an anti-inflammatory and insulin-sensitizing effect (44), a possible relationship between interleukin-33 and adiponectin in metabolic syndrome patients was assessed (39). No significant difference in serum IL-33 levels was observed between controls and patients with metabolic syndrome, whereas those with metabolic syndrome had reduced adiponectin levels. However, higher IL-33 levels were observed in a subgroup of patients with low adiponectin levels who also exhibited fewer features of metabolic syndrome, leading the authors to suggest that a combined analysis of IL-33 and adiponectin may be a useful predictor of inflammatory status during the preliminary onset of metabolic syndrome.

In the study by Tang et al. (5), the correlation between serum IL-33 levels and metabolic phenotypes in adult subjects was examined, revealing that IL-33 levels were elevated in metabolically unhealthy overweight/obese individuals, whereas no significant difference was observed between healthy controls and metabolically healthy overweight/obese individuals. In contrast, the study by Hasan et al. (6) demonstrated a negative correlation between IL-33 and body mass index (BMI) and body weight in lean and overweight individuals, but not in individuals with obesity. In addition, their study revealed that in nondiabetic patients, IL-33 was associated with a protective lipid profile, whereas no such association was found in patients with diabetes.

Taken together, and as seen from the results of our meta-analysis, it currently remains difficult to draw any firm conclusions about the role of IL-33 in human metabolic disease. Although individual studies may give a certain insight into the levels of IL-33 present and how this correlates with specific disease states, as there is considerable interstudy heterogeneity in the manner in which the studies were conducted, a causal association with IL-33 as either a protective or inflammatory mediator of human disease has not yet been adequately established.

#### IL-33 and exercise.

IL-33 has been shown to have a positive role in regulating the thermogenic capacity of adipose tissue (41, 43) and presents an attractive target for interventions aimed at combating metabolic disease. A study into the effects of exercise in overweight individuals with T2D investigated acute and chronic responses in both fed and fasting states. This study demonstrates that both forms of training increased the levels of serum IL-33 (32), and the authors propose that these increases in IL-33 may be induced as an adaptive response to exercise-induced stress, potentially fostering an anti-inflammatory environment. Exercise training was also shown to improve glycolipid metabolism and elevate postexercise IL-33 levels in individuals with T2D (38). Increased levels of IL-33 were observed following both aerobic and resistance training. Coincident with increases in IL-33, decreased levels of the proinflammatory cytokine IL-18 were also observed. In addition, the effects of high-intensity interval training on IL-33 levels have also been explored, where it was noted that individuals with greater cardiorespiratory fitness had elevated IL-33 levels (36), indicating that exercise may promote an anti-inflammatory milieu, as exercise correlated with decreases in the proinflammatory cytokines IL-6 and TNFα, while increasing the anti-inflammatory cytokine IL-10. These studies together indicate that IL-33 production could be induced in response to exercise and was also observed at greater levels in more fit individuals. Suggesting that exercise, particularly cardiorespiratory fitness, is a crucial factor in improving immune health through altering levels of inflammatory factors present. As such, there may be significant benefits from exercise in individuals with T2D and metabolic disease outside of those that are induced due to weight loss alone.

#### IL-33 and bariatric surgery.

In addition to lifestyle modifications such as diet and exercise, bariatric surgery represents one of the most effective strategies to induce significant weight loss and combat metabolic disease (45). In a follow-up study of patients at 1-yr postbariatric surgery, no significant differences in the levels of IL-33 were observed, even though postbariatric surgery patient BMIs were substantially reduced. However, a significant decrease in the levels of soluble ST2 (sST2) that acts as a circulating decoy receptor for IL-33 was observed and was particularly evident in individuals who initially presented with T2D (35). In the study by Katsogiannos et al. (20), changes in cytokine and adipokine levels post-Roux-en-Y gastric bypass surgery were examined. Here, it was reported that patients with obesity, with or without diabetes, had higher baseline IL-33 levels compared with healthy controls. Patients were followed up at 6 mo postsurgery, where it was observed that both groups had significant increases in IL-33 levels, which was coincident with a significant reduction in BMI. These studies indicate that, unlike sST2, IL-33 levels do not significantly decrease after surgery, suggesting that while IL-33 may potentially serve as a marker of obesity-related inflammation, it appears to be relatively unaffected by weight loss induced by gastric bypass surgery. However, the mechanistic pathways underlying the lack of change in IL-33 levels remain unexplored and may be influenced by an ongoing inflammatory response that remains in the adipose tissue postsurgery, as seen by the continuation of an elevated level of serum inflammatory markers (20).

#### IL-33 and associated conditions.

A series of diverse studies examining the relationship between IL-33 and various disease states has provided valuable perspectives. Notably, de Lima Azambuja et al. (34) proposed that IL-33 may play a dual role in asthma and obesity—potentially inducing Th2 responses while concurrently providing protection against obesity-related inflammation. In patients with asthma with varying BMIs, lower IL-33 levels were found in individuals with obesity, although this difference was not statistically significant. Serum IL-33 levels were also assessed in a comparison of subjects with allergic asthma and nonallergic asthma, where IL-33 showed a positive association with allergic asthma. When these subjects were assessed based on BMI, there was also no association shown with either the obese asthma or nonobese asthma groups (46), together, signifying that IL-33 may not be the common mediator linking asthma and obesity.

The role of IL-33 in myocardial infarction (MI) (37) was also investigated, where a significant increase in IL-33 levels in patients with MI relative to controls was reported. Patients with MI exhibited eight- to ninefold increases in IL-33 levels during hospitalization and up to a 17-fold increase at 1-yr post-MI. In addition, the extent of cardiac fibrosis demonstrated an inverse relationship with IL-33 levels, supporting an antifibrotic role for IL-33.

A protective role for IL-33 in pregnancy has been suggested, as lower serum concentrations of IL-33 in patients with preeclampsia were observed, along with an inverse correlation to BMI (31). Here, IL-33 levels were elevated from the first to third trimester in all study groups. Lower levels of IL-33 were linked with an increase in the uterine artery pulsatility index, suggesting a connection between IL-33 and placentation. The authors noted that a reduction in IL-33 production may lead to a proinflammatory state, inducing placental insufficiency linked to preeclampsia development.

Focusing on the inflammatory landscape of T2D, Alfadul et al. (17) reported a positive correlation between circulating NLRP3 and IL-33, potentially implicating IL-33 in diabetes pathogenesis. Importantly, higher NLRP3 levels in a female group with prediabetes suggest a potential sex-specific association, adding complexity to the inflammatory landscape of T2D. Furthermore, the relationship between IL-33 and diabetic nephropathy was assessed, where decreased IL-33 levels in individuals with nephropathy were observed. Consequently, the authors proposed IL-33 to be a potential therapeutic target for this condition due to its role in regulating Th2 responses (19).

Although these collective findings offer insights into the multifaceted role of IL-33 in various aspects of metabolic health, inflammation, and the immune response, it is essential to approach these observations with caution. Without a clear definitive role of IL-33 in human disease pathogenesis, it is difficult to extrapolate the current findings into useful therapeutic approaches. The overview of studies measuring IL-33 levels provided here emphasizes the need for further research, especially considering the limited sample sizes of some studies, to validate and expand upon the observed associations and implications.

### Quantitative Assessment and Challenges in Interpreting Serum IL-33 Levels

#### Statistical analysis of serum IL-33 levels in individuals with obesity and T2D.

Our meta-analysis aimed to quantitatively assess the significance of serum IL-33 levels in individuals with obesity and T2D to determine whether there was an association with disease. The findings regarding IL-33 serum levels in both patients with T2D and nondiabetic individuals with obesity versus healthy controls are inconclusive. In the T2D group, there was a tendency toward lower IL-33 levels, but the results lacked statistical significance. The study from Singh et al. (21) significantly influenced the observed interstudy heterogeneity, introducing complexity into the interpretation. Similarly, the meta-analysis did not reveal a significant difference in IL-33 levels in nondiabetic individuals with obesity compared with to healthy controls. Substantial heterogeneity among studies underscores potential confounding factors, necessitating further targeted investigations. Notably, the exclusion of the study by Dorneles et al. (36) from the sensitivity analysis led to a substantial reduction in heterogeneity. Given that the study exclusively included male participants, this specific demographic characteristic may serve as a confounding factor influencing overall heterogeneity. Unfortunately, the limited number of studies available for inclusion in our meta-analysis limited our ability to perform a meta-regression analysis to comprehensively assess potential confounding factors. Furthermore, in line with the findings of Demyanets et al. (35), who identified sex differences in sST2 levels, our sensitivity analysis, excluding the study by Dorneles et al. (36), supported the idea that sex-related variations could influence the dynamics of sST2 and, consequently, the IL-33/ST2 pathway in the context of metabolic health. Notably, Alfadul et al. (17) observed a positive correlation with IL-33 in a female prediabetic cohort, corroborating the potential existence of a sex-specific association. In addition, Tang et al. (5) suggested that sex-specific factors, such as menstrual cycle and sex hormone levels, may exert a significant influence on the association between IL-33 and metabolic disease. These collective findings indicate the need for further dedicated investigations into the relationship between IL-33 levels and sex.

#### Challenges in IL-33 quantification and the importance of standardization.

The observed disparities in IL-33 serum levels among healthy control groups in various studies highlight a significant issue—the diversity in employed measurement assays. Hasan et al. (6) used the DuoSet ELISA (detection range: 23.4–1,500 pg/mL) and found serum IL-33 detectable in only 32% of subjects, with 67% excluded due to levels being below the detection limit of the assay. Similarly, Singh et al. (21) used the same kit while others used ELISA kits with varying limits of detection: 15.6–1,000 pg/mL for the CUSABIO ELISA (5, 33) and 7.8–500 pg/mL for the Enzo Life Sciences (18). In contrast, de Lima Azambuja et al. (34) used Milliplex (minimum detectable concentration: 1.2 μg/mL), introducing a potential bias in reported levels and emphasizing the need for standardization in protocols and measurement assays. In addition, the establishment of a common method for reporting serum IL-33 data is crucial. Our analysis, covering studies using different metrics—median and geometric mean—indicates the need for a standardized reporting method to enhance comparability and interpretability across studies.

### Study Limitations

Tests for publication bias (Egger’s or Begg’s) were not conducted due to the small number of studies in each group (<10), limiting their power (47). Similarly, the meta-regression analysis lacked sufficient studies in comparison groups (5 for T2D patients vs. healthy controls and 6 for individuals with obesity vs. healthy controls) falling below the recommended minimum of 10 (48). These constraints highlight the need for cautious interpretation and emphasize the importance of future research to address these methodological limitations. Although the robustness demonstrated in the sensitivity analyses strengthens our study, the variations in effect sizes and heterogeneity observed limit the overall understanding of the role of IL-33.

### Conclusions

Our analyses indicate that the current evidence is insufficient to reliably determine a direct association for IL-33 with changes in inflammatory conditions in metabolic disorders. Here, meta-analysis does not provide conclusive evidence of significant differences in IL-33 serum levels between individuals with T2D or nondiabetic obesity and healthy controls. These findings underscore the need for improved serum IL-33 measurement methods and for further investigations with larger and more diverse study populations. The results presented in this systematic review and meta-analysis contribute to refining the knowledge about the relevance of IL-33 in individuals with obesity and T2D, guiding future research and enriching the broader discourse on therapeutic targets for metabolic disorders.

## DATA AVAILABILITY

The datasets supporting the conclusions of this article are included within the article and its additional files.

## SUPPLEMENTAL MATERIAL

10.6084/m9.figshare.25663797.v1 Supplemental Files S1–S3: https://doi.org/10.6084/m9.figshare.25663797.v1.

## GRANTS

Ghalia Missous is supported by the NPRP Award NPRP11S-0122-180359 from the Qatar National Research Fund (a member of The Qatar Foundation).

## DISCLAIMERS

The statements made herein are solely the responsibility of the authors.

## DISCLOSURES

No conflicts of interest, financial or otherwise, are declared by the authors.

## AUTHOR CONTRIBUTIONS

G.M. and N.V.P. conceived and designed research; performed experiments; analyzed data; interpreted results of experiments; prepared figures; drafted manuscript; edited and revised manuscript; approved final version of manuscript.
